# Molecular characterization reveals the complexity of previously overlooked coral-exosymbiont interactions and the implications for coral-guild ecology

**DOI:** 10.1038/srep44923

**Published:** 2017-03-30

**Authors:** H. Rouzé, M. Leray, H. Magalon, L. Penin, P. Gélin, N. Knowlton, C. Fauvelot

**Affiliations:** 1 UMR ENTROPIE (IRD, Université de La Réunion, CNRS), Laboratoire d’excellence-CORAIL; centre IRD de Nouméa, BPA5, 98848 Nouméa Cedex, New Caledonia, France; 2 National Museum of Natural History, Smithsonian Institution, Washington, DC 20013, USA; 3 UMR ENTROPIE (Université de La Réunion, CNRS, IRD), Laboratoire d’excellence-CORAIL; Université de La Réunion, 15 Boulevard René Cassin, CS 92003, 97744 Saint Denis, Reunion, Island, France

## Abstract

Several obligate associate crabs and shrimps species may co-occur and interact within a single coral host, leading to patterns of associations that can provide essential ecological services. However, knowledge of the dynamics of interactions in this system is limited, partly because identifying species involved in the network remains challenging. In this study, we assessed the diversity of the decapods involved in exosymbiotic assemblages for juvenile and adult *Pocillopora damicornis* types α and β on reefs of New Caledonia and Reunion Island. This approach revealed complex patterns of association at regional and local scales with a prevalence of assemblages involving crab-shrimp partnerships. Furthermore, the distinction of two lineages in the snapping shrimp *Alpheus lottini* complex, rarely recognized in ecological studies, reveals a key role for cryptic diversity in structuring communities of mutualists. The existence of partnerships between species that occurred more commonly than expected by chance suggests an increased advantage for the host or a better adaptation of associated species to local environmental conditions. The consideration of cryptic diversity helps to accurately describe the complexity of interaction webs for diverse systems such as coral reefs, as well as the functional roles of dominant associated species for the persistence of coral populations.

Mutualistic interactions are diverse, widespread and central to the structure and function of both terrestrial and marine ecosystems^1^,^2^,^3^. Tropical coral reefs, one of the most diverse ecosystem on Earth^4^, are particularly renowned for depending on complex networks of mutualistic relationships for their establishment and maintenance^3^,^5^. The collection of partnerships of scleractinian corals with other organisms represents one of the most complex interactive networks, in which multiple endo- (*in situ*) and exo- (*ex situ*) mutualistic species promote the growth, survival and reproduction of the coral host, which in turn provides crucial food and shelter. While the association between corals and their endosymbiotic zooxanthellae is the best studied^5^,^6^,^7^, the importance of the partnerships between corals and larger epifaunal macro-invertebrates^8^, mostly represented by decapod crustaceans^8^,^9^ is increasingly recognized. These understudied coral dwellers, also called exosymbionts, are either obligate or facultative (i.e., opportunistic) associates^10^.

Some obligate exosymbionts are well known for their key contribution to coral host survival, among the most notable being the crabs in the genus *Trapezia* and the snapping shrimp *Alpheus lottini* (Guérin Méneville 1829). These exosymbionts are both exclusively associated with pocilloporid corals^8^,^11^,^12^ and provide various cleaning^11^,^13^, physiological^14^ and defense services^12^,^15^,^16^ to their host, such as the removal of sediments and the deterrence of large corallivorous predators (e.g., *Acanthaster planci*).

Coral reefs are also known for the prevalence of cryptic species^17^, and corals with their mutualistic partners, both endosymbionts and exosymbionts, are no exception in this regard. In the case of the crustacean symbionts of pocilloporid corals, all members of the association include complexes of cryptic species. The best understood of these are the 22 described species of *Trapezia* crabs, whose distinctive color patterns and tendency to live in male female pairs can make identification of species less challenging. Identifications are mainly based on color and/or color patterns of the carapace, as well as, in a few cases, behavior^18^, but some similar species can be difficult to distinguish because of possible phenotypic plasticity^19^,^20^ or because of more ambiguous distinguishing features at the juvenile stage^8^,^16^. Similarly, cryptic species of *A. lottini* are likely, based on correlated genetic and color pattern differences^21^,^22^ and male-female pairing on corals, but because the clades have not been formally described, they are not routinely distinguished in ecological studies^8^,^11^,^23^. The most challenging are the coral hosts themselves, because of widespread phenotypic plasticity in response to environmental conditions^24^, the slow evolution of mitochondrial DNA (e.g.^25^), and the possibility of interspecific hybridization^26^ in corals generally. This is true in particular for members of the Pocilloporidae, despite the fact that some species have been used as a coral version of a ‘laboratory-rat’ for many years. However, progress in defining evolutionary lineages is being made^27^. For example, the so-called *P. damicornis* species, long recognized to be phenotypically plastic^28^, has recently been found to consist of at least three evolutionary lineages, including types α (*P. damicornis sensu*^27^) and β (*P. acuta sensu*^27^).

Recognizing this cryptic diversity is essential for understanding how these mutualisms work, and how evolutionary events, environmental factors and anthropogenic pressures may shape or threaten the ecological persistence of these partnerships^29^,^30^. Several species may co-occur and interact within a single host, leading to preferential patterns of associations with potential inhibitory or synergistic effects on the fitness of the host^31^. Patterns of co-occurrence of exosymbiont associations may in turn be contingent upon the host phenotype or genotype^32^,^33^,^34^. Recent evidence suggests that the exosymbiont species are not functionally equivalent in terms of the benefits they provide^35^.

In this context, the primary objective of the present study was to describe patterns of association among *P. damicornis* types α and β (*sensu*^27^; note that we used this designation because it better reflects the phylogenetic proximity between the two clades) and their decapod exosymbionts in two distinct biogeographic regions, the understudied South Western Indian Ocean (Reunion Island, RI), described as an evolutionary hotspot^36^, and the South Western Pacific Ocean (New Caledonia, NC), a region of interest for its high biodiversity^37^. At each location, surveys took place at a site with high impacts (HI) and low impacts (LI) from human populations. We used DNA barcoding to identify coral host species and delineate cryptic lineages of exosymbionts. We then analyzed patterns of co-occurrence among species involved in this mutualistic network in order to evaluate key exosymbiotic assemblages for the coral host.

## Results

### Species characterization

#### Coral host identification and colony sizes

We collected a total of 234 colonies, among which 88 and 146 were identified molecularly as *P. damicornis* types α and β, respectively (hereafter referred to α and β colonies; Table 1). In New Caledonia, higher proportions of *P. damicornis* type α were observed among both adults (81%) and juveniles (69%), and almost all type β colonies came from high impact sites (NC-HI, Table 1). In Reunion Island, only *P. damicornis* type β was found. Within type β, based on open reading frame (ORF) sequences, we observed no genetic differentiation between Reunion Island and New Caledonia or between the two sites at Reunion Island (Fig. S1).

Among the 234 colonies, 114 were juveniles (RI: N = 56; NC: N = 58) and 120 were adults (RI: N = 61; NC: N = 59). The planar areas calculated for selected juvenile colonies (types α and β) were not significantly different among the four sites (one-way ANOVA: F(3,100) = 2.22, *P* = 0.09; NC: mean ± standard error: 20 ± 1.4 cm^2^ at NC-LI and 25 ± 2.5 cm^2^ at NC-HI; RI: mean ± standard error: 23 ± 2.5 cm^2^ at RI-LI and 17 ± 1.9 cm^2^ at RI-HI). Furthermore, planar areas of juvenile colonies α and β, simultaneously present at NC-HI, were not significantly different (Kruskal-Wallis: df = 1, χ^2^ = 3.389, *P = *0.07). In contrast, planar areas of selected adult colonies were significantly different among sites (one-way ANOVA: F(3,102) = 165.7, *P* < 0.001), with colonies from New Caledonia being larger (mean ± standard error: 601 ± 22.6 cm^2^ at NC-LI and 416 ± 11.5 cm^2^ at NC-HI) than those from Reunion Island (median ± standard deviation: 297 ± 25.8 cm^2^ at RI-LI and 100 ± 5.9 cm^2^ at RI-HI). At NC-HI, adult coral colonies α versus β exhibited no significant differences in their planar areas (Kruskal-Wallis: df = 1, χ^2^ = 3.39, *P* = 0.96).

#### Exosymbiont identifications

**Figure 1:**
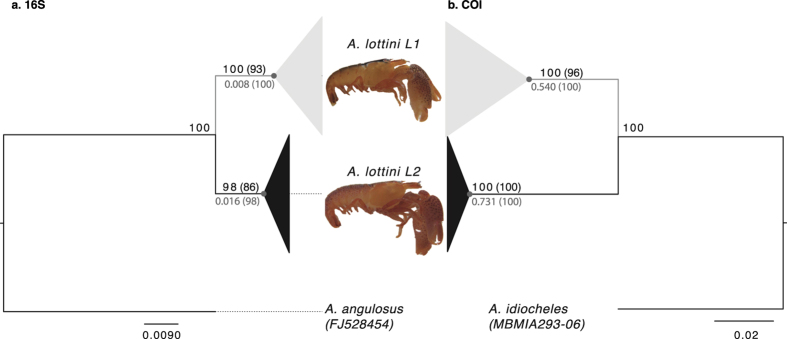
Phylogenetic tree of *Alpheus lottini* sequences derived from Bayesian approaches using 16S (**a**) and COI (**b**) partial genes. Bayesian posterior probabilities (PP; first values) and Maximum Likelihood bootstrap support values (BS; in brackets) are presented in black. PP and BS values corresponding to single putative species recognized by the PTP analyses are presented in grey.

In total, 162 specimens of the snapping shrimp *A. lottini* were collected. Phylogenetic analyses of both 16 S and COI sequences identified two highly supported divergent lineages (PP > 97%, BS > 85%; Fig. 1; Table 2). Net nucleotide divergence between these lineages was 15.2% for the COI gene, and 7% for the 16S gene. Lineage L1, characterized by a complex genetic structure (Fig. 1), occurred in both New Caledonia and Reunion Island, while lineage L2 was exclusively found in New Caledonia. Looking *a posteriori* at the color patterns of the two lineages, we observed that, while both exhibited a general orange color, the midline of the cephalothorax and abdomen of the L1 specimens presented a solid black band whereas lineage L2 displayed red dots down the midline (Fig. 1).

Based on all these results, snapping shrimps *A. lottini* are considered in all subsequent analyses as two genetically distinct lineages, called *A. lottini* L1 and L2, which based on studies of *Alpheus* species separated by the Isthmus of Panama^38^ are almost certainly distinct species that diverged millions of years ago.

**Figure 2:**
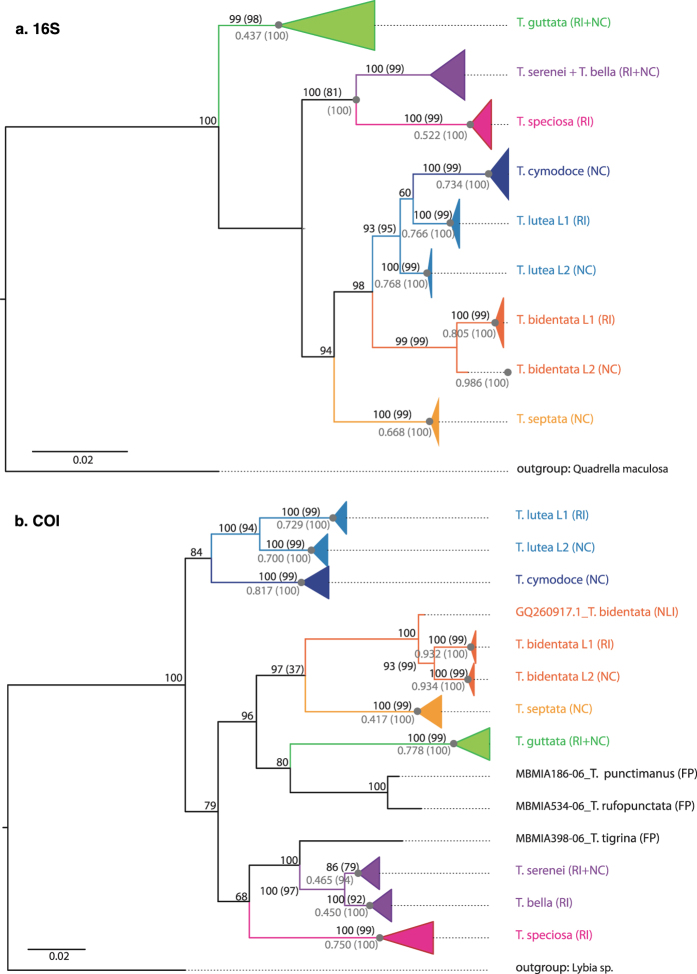
Phylogenetic tree of *Trapezia* sequences derived from Bayesian approaches using 16S (**a**) and COI (**b**) partial genes. Bayesian posterior probabilities (PP; first values) and Maximum Likelihood bootstrap support values (BS; in brackets) are presented in black. PP and BS values corresponding to single putative species recognized by the PTP analyses are presented in grey. Locations are coded as follows: RI for Reunion Island, NC for New Caledonia, FP for French Polynesia and NLI for Line Islands.

We collected a total of 332 *Trapezia* crabs which, based solely on morphological traits^39^, were identified as belonging to eight morphospecies (Fig. S2): *T. guttata* (Rüppel 1830), *T. serenei* (Odinetz 1984), *T. bella* (Dana 1852), *T. speciosa* (Dana 1852), *T. cymodoce* (Herbst 1801), *T. lutea* (Castro 1997), *T. bidentata* (Forskal 1775) and *T. septata* (Dana 1852). However, the phylogenetic analysis of a subset of individuals (at least one specimen per heterosexual pair and all juveniles; Table 2) revealed nine distinct supported clades based on 16S (N = 183, Fig. 2a) and ten based on COI (N = 163, Fig. 2b) (outgroup and GenBank/BOLD references excluded), which were also identified as distinct with bPTP models (Fig. 2).

Phylogenetic analyses of both COI and 16S genes revealed two highly supported monophyletic groups within the *T. lutea* clade (L1, L2), as well as two others within the *T. bidentata* clade (L1, L2); in both cases, these were considered as two distinct putative species with the bPTP models (Fig. 2). Also in both cases, the lineages were geographically partitioned: *T. lutea* L2 and *T. bidentata* L2 occurred in New Caledonia while *T. lutea* L1 and *T. bidentata* L1 occurred in Reunion Island (Fig. 2). Net nucleotide divergences (*T. bidentata* lineages: 2.1 and 2.5% for 16S and COI, respectively; *T. lutea* lineages: 2.6 and 4.4% for 16S and COI, respectively; Table S1) were comparable to what we observed between morphologically defined species both in allopatry or in sympatry (Table S1). However, no diagnostic color patterns specific to the genetic lineages within *T. lutea and T. bidentata* could be found (Fig. S2).

The discrepancy between the two markers in numbers of distinct putative species was due to inconsistent differentiation between two sympatric described species, *T. serenei* (RI and NC) and *T. bella* (RI) that mate assortatively. Although these taxa can be clearly distinguished by color patterns (Fig. S2), the 16S gene showed a very low level of genetic divergence (0.4%, Table S1) and branch support (PP: 84%; BS: 66%, respectively), in contrast to the COI gene (genetic difference: 1.4%; PP: 100%; BS: 97%). This inconsistency was also obtained with bPTP models, which delimited two distinct putative species based on the COI phylogeny, but only one putative species based on the 16S phylogeny (Fig. 2).

Based on these results of genetic distinction and/or color patterns, ten distinct species/lineages of *Trapezia* crabs are considered in all subsequent analyses: *T. guttata, T. speciosa, T. serenei, T. bella, T. cymodoce, T. lutea* L1 and L2, *T. bidentata* L1 and L2 and *T. septata*.

### Community composition of exosymbiont lineages

**Figure 3:**
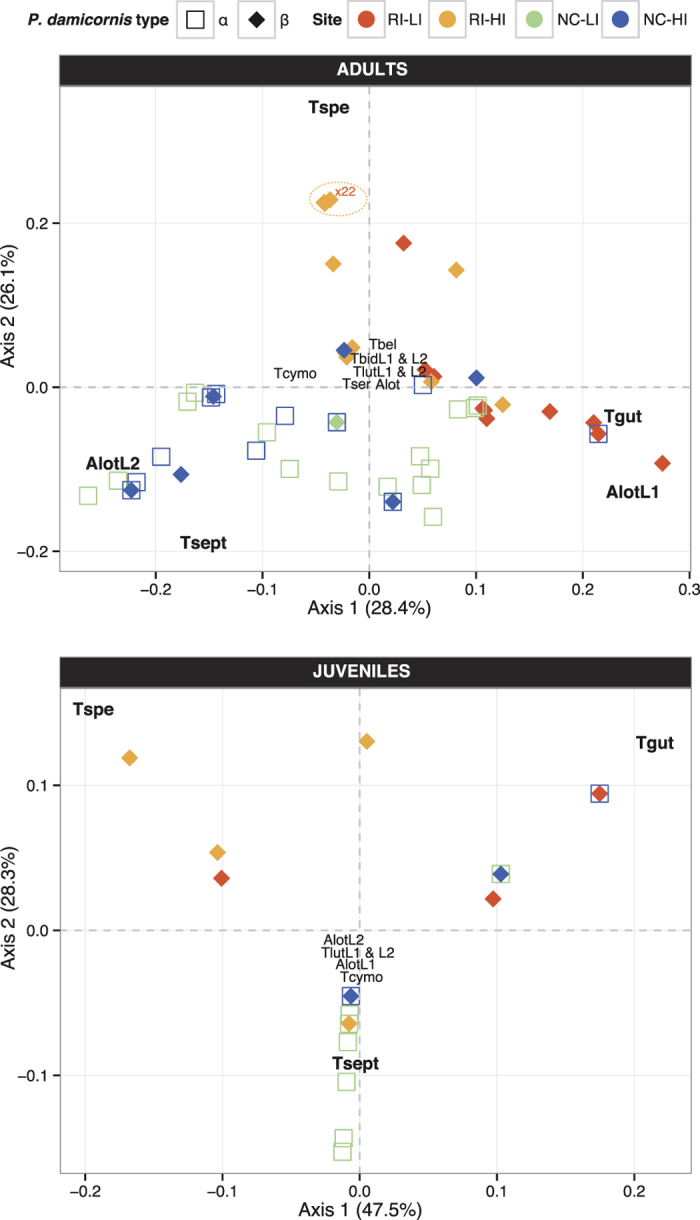
Factorial Correspondence Analysis (FCA) based on abundance of different *Trapezia* and *Alpheus lottini* species with *Pocillopora damicornis* types α versus β from Reunion Island and New Caledonia at adult (top) versus juvenile (bottom) stages. Exosymbionts species are denoted as follows: *Trapezia speciosa* (Tspe), *T. guttata* (Tgut), *T. septata* (Tsept), *T. cymodoce* (Tcymo), *T. bidentata* L1 (TbidL1) and L2 (TbidL2), *T. bella* (Tbel), *T. serenei* (Tser), *T. lutea* L1 (TlutL1) and L2 (TlutL2) for crabs, and *Alpheus lottini* L1 (AlotL1), *A. lottini* L2 (AlotL2) or non-identified (Alot) for shrimps. Species codes in bold represent the significant variables on axes 1 and/or 2 of the FCA analysis.

Factorial analysis (Fig. 3) showed compositional differences among communities of exosymbionts collected in *P. damicornis* α and β colonies according to biogeographic region (Indian versus Pacific Ocean) and sites (RI-LI, RI-HI, NC-LI and NC-HI). Three trapeziid crabs species (*T. speciosa, T. septata* or *T. guttata*) had a strong influence on the structure of exosymbiont communities, especially in adult corals.

Differences in community composition between adult coral hosts α versus β were significant when all colonies from New Caledonia and Reunion Island were considered (ANOSIM: R = 0.3814, *P* = 0.001) (Fig. 3). Statistical tests also indicated significant differences in composition between β hosts from New Caledonia and Reunion Island (ANOSIM: R = 0.3999, *P* = 0.001), but not between α versus β hosts from New Caledonia (ANOSIM: N_α_ = 48 and N_β_ = 11, R = −0.032 *P* = 0.63). Exosymbiotic communities were not significantly different between the two reef sites of New Caledonia (ANOSIM: R = 0.014, *P* = 0.28), but they differed significantly between the two sites at Reunion Island (ANOSIM: R = 0.688, *P* = 0.001). Five key species among the ten identified (*A. lottini* L1, *A. lottini* L2, *T. guttata, T. septata*, and *T. speciosa*; Fig. 3) drove these differences (Table S2). The first axis of the FCA, accounting for 28.4% of the total variance, discriminated communities characterized by *T. guttata* and *A. lottini* L1 (mainly observed in α colonies from NC-HI and β colonies from RI-LI) from communities characterized by *T. septata* and *A. lottini* L2 specific to New Caledonian sites (NC-LI and NC-HI mainly composed of α colonies)*. Trapezia septata* was the most prevalent species in α colonies (67%, Table 3) in New Caledonia, either in association with one pair of *A. lottini* L2 (23%) or with one pair of *A. lottini* L1 (21%), or as a unique species pair of exosymbionts (6.3%). Moreover, *T. septata* appeared to be the most gregarious species as it co-occurred with several other trapeziid species (*T. guttata, T. cymodoce, T. serenei* or *T. lutea* L2; Table 3). In contrast, *T. guttata* observed in both studied regions was found preferentially associated with different exosymbiotic species depending on the island: *T. guttata* (Table 3) mainly occurred with a pair of *A. lottini* L1 (68% of colonies harboring *T. guttata*), or living as a solitary pair (32% of colonies harboring *T. guttata*) in colonies from Reunion Island, while in New Caledonia it co-occurred with *A. lottini* L1 as often as with *A. lottini* L2 in β colonies. The second axis, representing 26.1% of the total variance, differentiated communities characterized by the presence of *T. speciosa,* which was typically found in colonies from RI-HI at Reunion Island (Table 3). This crab species was mostly observed by itself (85% of total communities including *T. speciosa*; Table 3) and occasionally in association with *A. lottini* L1 (N = 3).

**Figure 4:**
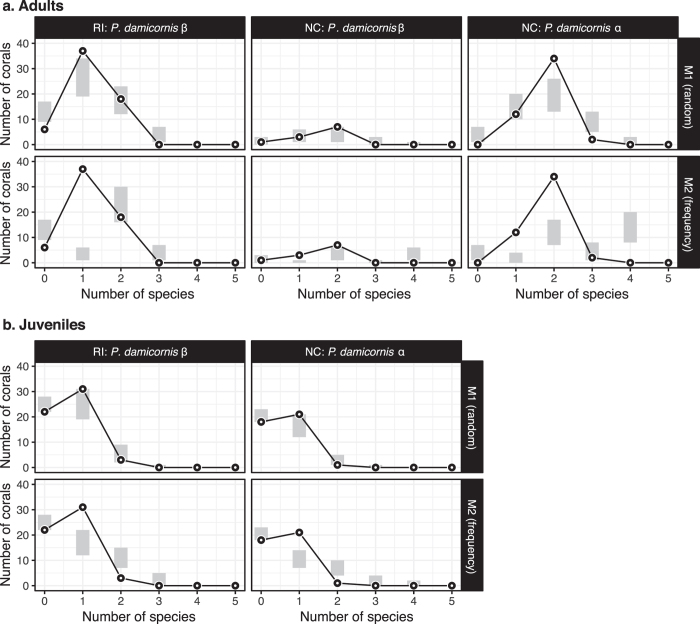
Number of exosymbiotic species among the five key species co-occurring within coral colonies: (**a**) adults of *Pocillopora damicornis* type β from Reunion Island (RI) and from New Caledonia (NC), and of *P. damicornis* type α from New Caledonia (NC), and (**b**) juveniles of *P. damicornis* type β from Reunion Island (RI) and type α from New Caledonia (NC). Black circles linked with a black line represent the observed data, while grey rectangles represent the 95% confidence intervals estimated from 10,000 randomly simulated communities generated with the unconstrained null model M1 and the ‘frequency’ constrained model M2 (see methods for a description of the models).

About half of *P. damicornis* juvenile colonies did not host any exosymbiont, regardless of location or host species type (α: 40% and β: 49%; Figs 3 and 4, Table 3). However, when present (RI: N_RI-LI_ = 18, N_RI-HI_ = 19 and NC: N_NC-LI_ = 23 and N_NC-HI_ = 2), exosymbiont communities were significantly different among the three reef sites where numbers were large enough to be analyzed (RI-HI, RI-LI and NC-LI; ANOSIM: R = 0.2782, *P* = 0.001), as well as between groups of juvenile β colonies from both sites of Reunion Island (RI-LI versus RI-HI; ANOSIM: R = 0.1287, *P* = 0.02). Three trapeziid crab species (*T. speciosa, T. guttata* and *T. septata*) played a key role in the community composition of juvenile corals (Table S2), and mainly occurred by themselves (Figs 3 and 4; Table 3). The first axis of the FCA, explaining 47.5% of the total variance, discriminated communities characterized by the two most prevalent species (*T. speciosa* and *T. guttata*) (Table 3). The communities shaped by the presence of *T. speciosa* were prevalent in juvenile hosts of type β from Reunion Island (64%; N_RI-HI_ = 16 and N_RI-LI_ = 8; Table 3), while those characterized by the presence of *T. guttata* were widely distributed on reefs of both Reunion and New Caledonia and in both host types: α (63%) and β (RI: 30% and NC: N = 1 colony; Table 3). The second axis of the FCA, explaining 28.3% of the total variance, differentiated exosymbiotic communities composed of crabs *T. septata*, the second most abundant species found in association with juvenile *P. damicornis* type α in New Caledonia (N_NC-LI_ = 6 and N_NC-HI_ = 0). *Trapezia speciosa, T. guttata* and *T. septata* co-occurred in rare cases but they were never recorded with *T. bella, T. serenei, T. lutea* L1 and L2 or *T. bidentata* L1 and L2.

### Species interactions

Observed patterns of association among the five key species revealed by the FCA were compared to their null distributions. These were generated with an unconstrained model (M1, which assumes that all species are able to colonize equally any coral host), a ‘frequency’ constrained model (M2, which assumes that some species are naturally able to colonize more corals than others), and a ‘richness’ constrained model (M3, only used in the analysis of species co-occurrence, which assumes that some corals are naturally more suitable to host different numbers of species).

More than half (61%) of adult colonies from Reunion Island (all type β) harbored only one of the five key species (Fig. 4a), a proportion significantly higher than expected by chance (observed: 37 colonies; 95% confidence interval (CI): 19–34 colonies for M1 and 1–6 colonies for M2). The frequency of corals occupied by a single species was particularly high at the RI-HI site (Table 3). In New Caledonia, the diversity of exosymbionts found in type β adult colonies did not differ from the null expectations (Fig. 4a; M1 and M2). On the other hand, type α adult colonies displayed a combination of two of the five key species in 34 out of 48 adult colonies, a proportion much higher than expected by chance (95% CI: 13–26 colonies for M1 and 7–17 colonies for M2; Fig. 4a). The number of adult colonies inhabited by a single species (observed: 12 colonies) overlapped with the null distributions obtained with the unconstrained model (95% CI: 10–20 colonies for M1), but not with the frequency constrained model (95% CI: 0–4 colonies for M2). Only 4% of adult colonies, all type α, contained three of the five key species, and no coral harbored four or five key species (Fig. 4a). Juvenile corals belonging to *P. damicornis* type β from Reunion and type α from New Caledonia, in contrast, did not harbor more than one exosymbiont species (except one juvenile α from NC and three juveniles β from RI, Table 3), an observed distribution overlapping the null distributions (Fig. 4b) of the unconstrained model M1 but not the null distributions of the constrained model M2 (Fig. 4b). Indeed, when constraining species frequency in randomizations (M2), the observed proportions of juvenile corals inhabited by a single species was significantly higher than expected by chance in New Caledonia (observed: 21 colonies; 95% CI: 7–14 colonies) and in Reunion Island (observed: 31 colonies; 95% CI: 12–22 colonies). Only one out of the 18 juvenile β colonies from New Caledonia was inhabited by exosymbionts (one *T. guttata*), preventing any comparison with null distributions.

**Figure 5:**
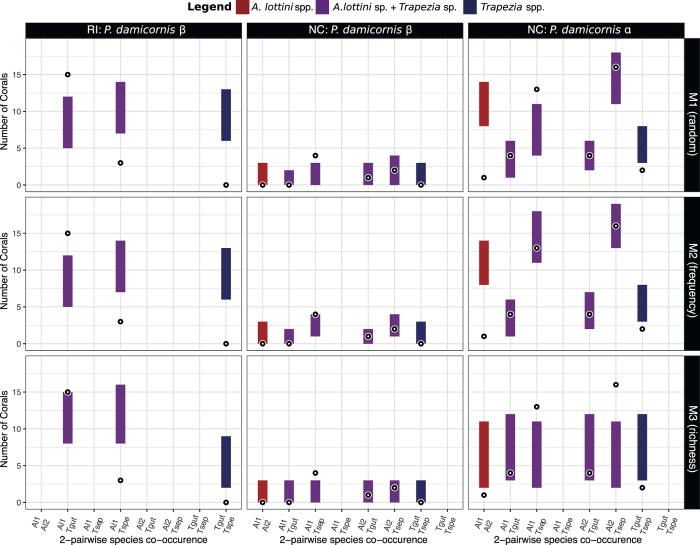
Interspecific pairwise co-occurrence of five exosymbiotic key species in adult *Pocillopora damicornis* type β from Reunion Island (RI) or from New Caledonia (NC), and type α from New Caledonia (NC). Black circles represent the observed data. Colored rectangles represent the 95% confidence intervals from 10,000 randomly simulated interactions between shrimp-shrimp (red), crab-shrimp (purple) or crab-crab (blue) assemblages generated with the unconstrained null model M1, the ‘frequency’ constrained model M2 and the ‘richness’ constrained model M3 (see methods for a description of the models).

Observed patterns of species co-occurrence in adult colonies compared to their null distributions generated with unconstrained (M1) and constrained (M2 and M3) models showed contrasting results between host species and locations (Fig. 5). Type β colonies inhabited by two species of exosymbionts at Reunion Island (30% of the coral population) were characterized by the presence of two shrimp-crab combinations, both deviating from null models of random association (Fig. 5). The first crab-shrimp association, *T. speciosa* with *A. lottini* L1, was significantly under-represented in the three null models (observed: 3 colonies; 95% CI: 7–14 colonies for M1 and M2 and 8–16 colonies for M3), while the second, *T. guttata* with *A. lottini* L1, was significantly over-represented in comparison to the null distributions obtained with models M1 and M2 only (observed: 15 colonies; 95% CI: 5–12 colonies for M1 and M2 and 8–15 colonies for M3; Fig. 5). Moreover, not a single colony hosted *T. guttata* and *T. speciosa* together, the two most common *Trapezia* species in the Reunion Island survey, which is far below what would be expected by chance based on all three null models (95% CI null distributions: 6–13 colonies for M1 and M2, and 2–9 colonies for M3). Among the five key species, only *T. speciosa* mostly occurs by itself in a proportion significantly higher than distributions of the three null models (observed: 24 colonies; 95% CI: 6–14 colonies for M1, 7–15 colonies for M2 and 7–18 colonies for M3; Fig. S3). Type β colonies from New Caledonia (Fig. 5) also harbored shrimp-crab combinations. However, unlike corals from Reunion Island, *T. guttata* never co-occurred with *A. lottini* L1 or did not show any preferential association with *A. lottini* L2 (observed distributions overlap with the null distributions of the three null models, Fig. 5). The most prevalent association observed was *T. septata* with *A. lottini* L1 (observed: 4 colonies, Fig. 5), a proportion slightly higher than the null distributions of models M1 and M3 (95% CI: 0–3 colonies for both models) but overlapping with null distributions of model M2 (95% CI: 1–4 colonies). The same preferential association between *T. septata* and *A. lottini* L1 was recorded in type α colonies based on models M1 and M3 (observed: 13 colonies; 95% CI null distributions: 4–11 colonies for M1 and 2–11 colonies for M3; Fig. 5). However, the co-occurrence of *T. septata* with *A. lottini* L2, the most frequently observed combination, did not deviate from predictions of the null models M1 and M2 (observed: 16 colonies; 95% CI: 11–18 and 13–19 colonies for M1 and M2, respectively) but deviate from those obtained with the null model M3 (95% CI: 2–11 colonies). In addition, type α colonies often hosted *T. guttata* with one of the two lineages of *A. lottini* but their co-occurrence did not deviate from the three null models (Fig. 5). The co-occurrences of *A. lottini* L2 with *A. lottini* L1 (observed: 1 colony; 95% CI null distributions: 8–14 colonies for M1 and M2, and 2–11 colonies for M3) and *T. guttata* with *T. septata* (observed: 2 colonies; 95% CI null distributions: 3–8 colonies for M1 and M2, and 3–12 colonies for M3) were observed less frequently than expected by chance in corals for all the null models (Fig. 5).

## Discussion

Genetic data confirmed the ubiquity of sibling species among crustaceans (e.g.^40^,^41^,^42^,^43^,^44^,^45^,^46^). *Alpheus lottini*, currently considered in ecological studies as a single species, constituted two highly divergent genetic lineages that mate assortatively and exhibited corresponding differences in color pattern, indicating that they are almost certainly separate species. These two lineages, previously detected in allopatry and sympatry in other studies^21^,^22^, were also found in sympatry in New Caledonia. However, the two distinct lineages were rarely found within the same coral host, indicating habitat partitioning typical of a micro-allopatric distribution. Similarly, molecular analyses revealed the presence of previously unrecognized, allopatric cryptic taxa within two morphospecies of *Trapezia (T. lutea* and *T. bidentata*) that have genetic divergences comparable to those between closely related described sympatric and/or allopatric species (Table S1) and that mate assortatively. While we did not observe any conspicuous color pattern differences (Fig. S2), differentiation of these potentially cryptic species could be explored in the future using morphometric measurements or more detailed color pattern analysis. With all these observations it seems unlikely, but not excluded, that these divergent clades may highlight strong geographically based genetic structure within both species. On the other hand, analyses based on 16S sequences did not support a genetic distinction between *T. bella* and *T. serenei,* despite a clear distinction of two monophyletic clades based on the COI marker, color patterns (Fig. S2 39) and mating pattern. Given the slower evolutionary rate of the 16S gene compared to COI gene (e.g.^47^), together with the low pairwise genetic difference in COI (1.4%), our findings suggest a recent speciation event between *T. bella* and *T. serenei*.

Molecular identification of surveyed hosts and exosymbionts enabled the detection of preferential associations that would otherwise have been missed. In particular, it revealed that each of the two genetically revealed lineages of *A. lottini* had different affinities with trapeziid crabs. For example in New Caledonia, while both *A. lottini* lineages were commonly found in association with *T. septata*, only the partnership with *A. lottini* L1 occurred more often than by chance (models M1 and M3, Fig. 5). These findings suggest that the lineages of *A. lottini* not only differ genetically but also in ecological traits that affect the strength of interactions with co-occurring trapeziid crabs.

Shrimp-crab associations were predominant in adult *P. damicornis* α and β colonies in both biogeographic provinces. This pattern is in accordance with behavioral observations that described resident crabs and snapping shrimps occupying slightly different portions of the coral host^48^, therefore minimizing competitive interactions^23^,^49^. Some authors have even reported positive interactions, with *A. lottini* physically cleaning the chelipeds of *T. cymodoce*^23^. On the other hand, both are highly territorial and exclude conspecific intruders that share similar ecological requirements^23^. Mechanical or chemical communication by direct contact with crabs might vary according to the exosymbiotic species involved. Furthermore, our survey showed that the species involved in crab-shrimp associations differed greatly between geographic provinces, mostly because several trapeziid species were restricted to only one locality (e.g., *T. speciosa* at Reunion Island and *T. septata* in New Caledonia) and because *A. lottini* L2 was absent from Reunion Island. Moreover, we found that patterns of association for a given species pair differed between New Caledonia and Reunion Island, highlighting differences in the strength of interactions at the regional scale. For example, *T. guttata* preferentially associated with *A. lottini* L1 in Reunion Island (null models M1 and M2; Fig. 5), while these two species co-occurred randomly (no deviation from the three null models) in New Caledonia.

Marked differences in community composition at the local scale, i.e., between sites within a region, were also detected. One of the most striking differences occurred at Reunion Island, where adult corals of the high impacted site were mostly inhabited by a single crab species (*T. speciosa*) in proportions higher than expected by chance (i.e., three null models; Fig. S3). The establishment of dominant multi-specific or mono-specific assemblages of mutualists can be generated by several ecological and/or evolutionary factors (reviewed in ref. 1). At this site, characterized by high sedimentation and eutrophication^50^, corals were smaller and with thicker branches than at the less impacted site. Therefore, one possible mechanism explaining the preponderance of monospecific assemblages may simply be the limited habitat space^23^. However, observed patterns of species interactions indicate that simple space limitation is not the main ecological mechanism involved. For example, the occurrence of *T. speciosa* with other key common species was significantly lower than expected (i.e. *A. lottini* L1 or *T. guttata*, Fig. 5) under the three null models whereas other sedentary coral-obligate species (e.g., *Paragobiodon* fishes, data not shown) sometimes co-occurred with *T. speciosa* in the same coral colony. However, the territorial behavior of *T. speciosa* against close competitors may lead to the exclusion of conspecifics and *A. lottini*, as suggested by studies highlighting differences in competitive and territoriality abilities^18^,^35^ among exosymbionts. The decrease of diversity within guilds of mutualists in response to interspecific competition is a general ecological process, particularly well documented in terrestrial ecosystems (e.g. plant-mycorrhizal mutualisms^51^). The chronic exposure to environmental stressors at this site may also affect the dynamics of interactions among exosymbionts, from mutualism to competition, as was previously observed during prolonged exposure to thermal stress^52^. Finally, ecological specialization to host morphology could also be part of the explanation as it has been observed in other systems, such as cryptic species of barnacles which preferentially associate with different *Millepora* morphospecies^33^. Regardless of the mechanisms involved, this represents a rare case where *Trapezia* species do not display any preferential partnership with a heterosexual pair of *A. lottini* shrimps^11^,^12^,^53^ or even with other congeners in adult coral hosts.

At the juvenile stage, about half of monitored coral colonies lacked exosymbionts, while the others were associated with a single juvenile trapeziid crab. Interestingly, *T. speciosa* crabs were dominant among juvenile coral colonies at the impacted reef site in Reunion Island, similarly to what was observed for adult corals. This suggests either high levels of self-recruitment in these crab populations (e.g.^54^) or recruitment failure of other crab species (e.g., *T. guttata*), most likely linked with unfavorable environmental conditions. Conversely, there was no relationship between community composition of exosymbionts in juvenile versus adult corals at the low impacted site in Reunion Island (RI-LI) or at either site in New Caledonia, all characterized by multispecific exosymbiotic communities in adult corals. One hypothesis for this result is that patterns of recruitment are highly variable through time, which translates into changes in species associations in adult corals. Alternatively, the establishment of multispecific partnerships may occur later during the ontogeny of the coral colony through migrations of adult shrimps and crabs^53^ or even through effective recruitment of other exosymbiotic species when living space becomes sufficient.

The absence of differences in exosymbiont community structure between sympatric *P. damicornis* types α and β in New Caledonia is another interesting ecological feature of mutualistic coral-exosymbionts partnerships that emerged from our results. It suggests that associated exosymbiotic communities are not strictly host specific, and that similar exosymbiont communities can be found in closely related coral species, which despite their different reproductive traits^55^, may not be completely reproductively isolated^26^. This preliminary assumption will need further investigation to be confirmed by obtaining more balanced sample sizes of types α and β from the same site. Furthermore, an effect of the host genet as a driver of exosymbiont community composition (e.g.^56^,^57^) among coral colonies type α or β cannot be assumed, since some coral clones (i.e., colonies with similar multi-locus genotypes) had different exosymbiotic communities (data not shown).

Variation in exosymbiotic community composition between regions and habitats likely translates into fitness differences among host populations, depending on ecological traits of the exosymbiont species involved. The ability of one heterosexual pair of crabs and shrimps to co-occur, while they both exclude congeners, represents a selective advantage to the coral host because the benefits provided to the host facing acute environmental stressors increase with the number and diversity of exosymbionts (e.g., sedimentation load^11^, predation^12^,^49^). Therefore, coral hosts associated with a single pair of *T. speciosa* in the high impacted site at Reunion Island may prove less resilient to future episodes of sedimentation or predator outbreak than hosts harboring multi-specific crab-shrimp communities. Alternatively, the association between the coral host and a single species of *Trapezia* could be more stable in a permanently disturbed environment than fragile crab-shrimp communities^52^ in which both species depend on each other^23^. Moreover, services provided to the host are dependent upon the species involved as well as the nature of the environmental stressor. For example, it was shown that the co-occurrence of *T. serenei* crabs and *A. lottini* shrimps had an additive effect on the removal of sediments, but they combined synergistically to better defend their coral host from corallivorous predators^12^. Because *A. lottini* has been considered as a single species in ecological studies to date, we do not know how the newly discriminated species might differ in their functional roles.

Our findings highlighted the complexity of coral-guild networks with preferential exosymbiotic associations that vary between sites and biogeographic regions. Ecological trade-offs for coral hosts that associate with mono- versus multi-specific exosymbiotic communities, as well as functional benefits of various species pairs including the cryptic taxa identified herein, remain poorly understood. Therefore, further research focusing on functional traits of key exosymbiont species, as well as on the nature of interactions (synergy versus antagonism) among them in the context of their sensitivity to various environmental conditions is critical. Understanding the geographical mosaic of exosymbiont species associations will shed light on how these communities affect the resistance and resilience of coral populations to ongoing environmental changes.

## Material and Methods

### Specimen collection and measurement

Colonies presenting *P. damicornis-*like morphology (identified a posteriori as types α and β *sensu* Schmidt-Roach *et al*.^27^) were collected by SCUBA or snorkeling in two distinct biogeographic regions: Reunion Island (RI) in the South Western Indian Ocean and New Caledonia (NC) in the South Western Pacific Ocean (Table 1). At each island, we selected two fringing reefs where juveniles and adults were abundant and that appeared to differ in the intensity of anthropogenic environmental stressors based on reports of sedimentation rates and/or nutrient concentrations (NC^58^,^59^,^60^ and and RI^50^). We chose ‘RI-HI’ in RI (21°20’S, 55°28’E) and ‘NC-HI’ in NC (near S^te^ Marie Bay, 22°18’S, 166°28’E) as high impacted sites, and ‘RI-LI’ in RI (lagoon channel, 21°5’S, 55°13’E) and ‘NC-LI’ in NC (22°15’S, 166°19’E) as low impacted sites. Since differences in coral size are known to affect the composition of exosymbiont communities^16^,^61^,^62^, we focused our sampling on two size classes of corals: juveniles colonies with diameter ≤5 cm^63^ and large adult colonies with diameter >10 cm. Numbers of adult and juvenile corals sampled at each site are summarized in Table 1. We measured the maximum diameter (L) and the maximum perpendicular diameter (l) of each coral colony to estimate *a posteriori* the planar area using the formula for an ellipse (S = π × L/2 × l/2). A plastic bag was placed over each colony to minimize the loss of exosymbionts prior to detachment from the substrate using a hammer and chisel. Individual colonies were then transported back to the laboratory. All *Trapezia* crabs and *A. lottini* shrimps were extracted by hand by fragmenting the colonies, identified to the species level using available morphological identification keys^39^, photographed alive and individually preserved in 80% ethanol. A small piece of the coral host sampled directly underwater was also preserved in 80% ethanol for subsequent molecular identification. All experimental protocols carried out on live material (corals and exosymbionts) were performed according to collection permits approved and obtained by the ‘Direction de l’Environnement, de l’Aménagement et du Logement de La Réunion’ (order n° 2013–23) and the ‘National Marine Reserve of Reunion Island’ as well as the Province Sud of New Caledonia (order n° 1315–2014/ARR/DENV).

### Molecular analyses

Host species identity was checked for all sampled colonies by amplifying the mitochondrial open reading frame (ORF) region using the FATP6.1 and the RORF primers^64^. Coral DNA (polyps + microorganisms) was extracted using the Gentra Puregene Tissue Kit (Qiagen, Germany). For crustaceans, a small piece of muscle tissue was sampled from each individual crab and shrimp for total genomic DNA extraction using the DNeasy Blood and Tissue kit (Qiagen, Germany). A portion of the cytochrome *c* oxidase subunit I (COI) gene and the 16S rRNA gene were amplified for each exosymbiont using universal primers (COI: LCO1490/HCO2198^65^ and 16S: 16Sar/16Sbr^66^).

All PCR reactions were performed in a final volume of 20 μL using the Type-It Microsat kit (Qiagen, Germany), following manufacturer recommendations and 1–10 ng of template DNA. Amplification conditions were as described in Schmidt-Roach *et al*.^55^ for ORF and Leray *et al*.^67^ for COI. The following temperature profile was used for the amplification of 16S: 95^◦^C for 5 min, followed by 35 cycles of 30 sec at 95^◦^C, 30 sec at 50^◦^C and 45 sec at 72^◦^C, followed by a final extension at 72^◦^C for 5 min. Both 16S and ORF amplicons were sent for sequencing in one direction to Genoscreen (Lille, France) and purified COI products to the Laboratory of Analytical of Biology of the Smithsonian National Museum of Natural History for sequencing in both directions.

Chromatograms were edited and aligned using MUSCLE implemented in Mega 6.06 and adjusted by eye in a single alignment per group and per marker. Publicly accessible databases (i.e., GenBank, BOLD) were searched for homologous sequences of *Trapezia* and *Alpheus*, as well as outgroup sequences, and these were added to previous alignments. For the hosts, the ORF sequences were aligned with those generated by Schmidt-Roach *et al*.^55^ as the reference (PopSet accession number: 413910611).

### Data analyses

Phylogenetic relationships among species were estimated independently per group and per marker using Bayesian Markov Chain Monte Carlo (MCMC) analysis (MrBayes 3.2.4). The best substitution model was determined using the Bayesian Information Criterion, as implemented in MEGA 6.06 (Table 2), across sites for 1 to 5 million generations. Bootstrap support values were also assessed from 2000 Maximum Likelihood (ML) replicate trees in MEGA 6.06. Phylogenetic trees were represented with both Bayesian posterior probabilities (PP) and ML bootstrap support (BS) values with FigTree 1.4.2. In addition, as a method for delimiting exosymbiont species, we used the Maximum Likelihood implementation of the Poisson Tree Processes model based on Bayesian phylogeny gene trees (bPTP^68^). The bPTP model uses the number of substitutions to simulate speciation and coalescent events, assuming that the number of substitutions is significantly higher between individuals belonging to different species than between conspecifics. The NEXUS trees were used as input data and submitted to the bPTP webserver (http://species.h-its.org/ptp/), but with reduced number of sequences in the *Trapezia* dataset, because PTP tends to overestimate the number of recognized species when there is uneven sampling of individuals per species^68^. In our *Trapezia* matrix, a large number of sequences were assigned to *T. guttata, T. speciosa* and *T. septata*, so we first removed identical sequences and pruned additional sequences that showed little variation in our dataset. The bPTP analyses were run without the outgroup (improving species delimitation) with 5 million MCMC generations with a burn-in of 25%, and other parameters with default values. All sequences were deposited in GenBank (accession numbers: for exosymbionts detailed in Table 2, and KY747218-KY747245 for *Pocillopora*).

Following identification of molecular lineages for both coral hosts and exosymbionts, similarities between communities of exosymbionts were visualized in two dimensions using a Factorial Correspondence Analysis (FCA) performed using the ade4 package in R 3.1.2. FCA uses a chi-square distance to measure similarities between communities without taking into account coral colonies with no observations. Compositional differences were tested between coral types (α versus β) and between locations (New Caledonia versus Reunion Island) using an analysis of similarity (ANOSIM) performed in R with the Vegan package^69^.

To further explore patterns of association for the five most common exosymbiont species, we compared observed patterns of species richness and co-occurrence in our survey to patterns expected by chance in randomly simulated communities. To mimic ecological patterns under various scenarios, we generated a set of three null models: (1) an ‘unconstrained’ model (M1) in which each of the five species was randomly assigned to corals; (2) a ‘frequency’ model (M2) in which the total number of occurrences of each species was fixed to account for the fact that some species are more common than others in communities; and (3) a ‘richness’ model (M3) (simulated for comparison to observed patterns of co-occurrence only) in which the total number of species per coral was fixed to consider the fact that some corals are naturally more suitable to host different numbers of species (e.g. larger corals can host more species of crab). The two constrained models were simulated in R using the Picante Package^70^. Each null model was generated separately depending on the biogeographic region (RI or NC) and/or the coral types (α or β) to test their effect on coral community structure and because they likely comprise distinct species pools. For each null model, the total number of coral hosts was fixed at 61 (RI all type β), 48 (NC, type α) and 11 (NC, type β) for adults and 56 (RI all type β) and 40 (NC all type α) for juveniles (Table 1), because these values corresponded to numbers of corals inhabited by at least one of these exosymbiont species. Ten thousand random communities were simulated for each null model, and the 95% confidence interval (CI) of the null distribution was estimated and then compared to our observed distribution. The hypothesis of random species association was rejected if observed values fell outside the 95% confidence interval. We tested for differences in planar areas of coral colonies between sites using analyses of variance (ANOVA), in case of homogeneity of variance, or with non-parametric Kruskal-Wallis tests on ranks.

## Additional Information

**How to cite this article:** Rouzé, H. *et al*. Molecular characterization reveals the complexity of previously overlooked coral-exosymbiont interactions and the implications for coral-guild ecology. *Sci. Rep.* **7**, 44923; doi: 10.1038/srep44923 (2017).

**Publisher's note:** Springer Nature remains neutral with regard to jurisdictional claims in published maps and institutional affiliations.

## Supplementary Material

Supplementary Information

## Figures and Tables

**Table 1:** Number of colonies of *Pocillopora damicornis* type α and β (*sensu* Schmidt-Roach *et al*. 2014^27^) monitored for their associated exosymbiotic communities at the adult and juvenile stages and from different sites at Reunion Island and New Caledonia.

Region	Island	Site	Stage	Type
Indian Ocean	Reunion	RI-LI	Adult	β: N = 30
			Juvenile	β: N = 31
		RI-HI	Adult	β: N = 31
			Juvenile	β: N = 25
Pacific	New Caledonia	NC-LI	Adult	α: N = 29, β: N = 1
			Juvenile	α: N = 30
		NC-HI	Adult	α: N = 19, β: N = 10
			Juvenile	α: N = 10, β: N = 18

**Table 2:** Sequence sets used for each crab and shrimp group with mtDNA 16 S and COI partial sequences.

Gene	Groups	Phylogenetic model	Genus	Species	Present study			External sequences			
					Number	Accession no.	Location	Accession no.	Library	Location	Citation
16 S	Shrimp	K2 + G	*Alpheus*	*A. lottini L1*	N = 38	KY746726-KY746763	NC, RI	—	—	—	—
		G = 0.21	*Alpheus*	*A. lottini L2*	N = 31	KY746764-KY746794	NC	KP725488.1	Genbank	Mayotte	[1]
			*Alpheus*	*A. angulosus*	N = 1	—	—	FJ528454	Genbank		[2]
										
	Crab	HKY + G	*Quadrella*	*Q. maculosa*	N = 1	—	—	FJ548954	Genbank		[3]
		G = 0.16	*Trapezia*	*T. guttata*	N = 50	KY747168-KY747217	RI, NC				
			*Trapezia*	*T. serenei*	N = 5	KY747063-KY747067	RI, NC				
			*Trapezia*	*T. bella*	N = 5	KY747058-KY747062	RI				
			*Trapezia*	*T. speciosa*	N = 57	KY747068-KY747124	RI				
			*Trapezia*	*T. cymodoce*	N = 8	KY747050-KY747057	NC				
			*Trapezia*	*T. lutea L1*	N = 5	KY747039-KY747043	RI				
			*Trapezia*	*T. lutea L2*	N = 6	KY747044-KY747049	NC				
			*Trapezia*	*T. bidentata L1*	N = 2	KY747036-KY747037	RI				
			*Trapezia*	*T. bidentata L2*	N = 1	KY747038	NC				
			*Trapezia*	*T. septata*	N = 43	KY747125-KY747167	NC				
										
COI	Shrimps	K2 + G + I	*Alpheus*	*A. lottini L1*	N = 46	KY746795-KY746840	NC, RI				
		G = 2.28	*Alpheus*	*A. lottini L2*	N = 32	KY746841-KY746872	NC, NC	MBMIA477-06	BOLD	FP	
		I = 0.68	*Alpheus*	*A. idiocheles*	N = 1			MBMIA293-06	BOLD	FP	
										
	Crab	GTR + G	*Lybia*	*L. sp. LP-2009*	N = 1	—	—	GQ260943	Genbank	NLI	[4]
		G = 0.25	*Trapezia*	*T. guttata*	N = 52	KY746954-KY747005	RI	GBCMD9223-13	BOLD	Australia	[5]
			*Trapezia*	*T. serenei*	N = 5	KY747031-KY747035	RI, NC	MBMIA599-06	BOLD	FP	
			*Trapezia*	*T. bella*	N = 5	KY747026-KY747030	RI				
			*Trapezia*	*T. speciosa*	N = 50	KY746873-KY746922	RI				
			*Trapezia*	*T. cymodoce*	N = 4	KY747018-KY747021	NC	HM751069	Genbank		[6]
			*Trapezia*	*T. lutea L1*	N = 5	KY747006-KY747010	RI				
			*Trapezia*	*T. lutea L2*	N = 7	KY747011-KY747017	NC				
			*Trapezia*	*T. bidentata L1*	N = 2	KY747022-KY747023	RI				
			*Trapezia*	*T. bidentata L2*	N = 2	KY747024-KY747025	NC				
			*Trapezia*	*T. bidentata L3*	N = 1	—	—	GQ260917.1	Genbank	Line Islands	DS
			*Trapezia*	*T. septata*	N = 31	KY746923-KY746953	NC	GBCMD9204-13	BOLD	Australia	[5]
			*Trapezia*	*T. tigrina*	N = 1			MBMIA398-06	BOLD	FP	DS
			*Trapezia*	*T. punctimanus*	N = 1			MBMIA186-06	BOLD	FP	DS
			*Trapezia*	*T. rufopunctata*	N = 1			MBMIA534-06	BOLD	FP	DS

The nucleotide substitution models used are indicated as follows: K2 for Kimura-2-parameter^71^, HKY for Hasegawa-Kishino-Yano^72^ and GTR for General Time Reversible^73^, with Gamma correction (G) and/or with a certain fraction of sites evolutionary invariable (+I). Locations are coded as follows: RI for Reunion Island, NC for New Caledonia, FP for French Polynesia and LNI for northern Line Islands.

**[1]**^74^**; [2]**^75^**; [3]**^76^**; [4]**^77^**; [5]**^9^**; [6]**^78^; DS direct submission.

**Table 3:** Composition of exosymbiotic communities and their frequencies in colonies of *Pocillopora damicornis* type α and β (*sensu* Schmidt-Roach *et al*. 2014) at the adult and juvenile stages and in different sites [low impacted (LI) versus high impacted (HI)] from Reunion Island (RI) and New Caledonia (NC).

	Assemblage			Site LI				Site HI			
Island	*Trapezia*	*A. lottini*	Additional	Adult		Juvenile		Adult		Juvenile	
				α	β	α	β	α	β	α	β
NC	T. septata*	A. lottini L1*	—	8		1		2	4		
			*T. serenei*	1							
			*T. serenei + A. lottini* (N = 3 juveniles)	1							
			*T. lutea L2*	1							
		A. lottini L2*	—	6				5	2		
			*T. cymodoce*	2							
			T. guttata*	2							
			*T. serenei*					1			
		—	—			5		3			
	T. guttata*	A. lottini L1*	—	1				3			
		A. lottini L2*	—		1			2			
		—	—			14			2	1	1
	*T. cymodoce*	A. lottini L2*	—	2							
			*T. lutea L2*	1							
			A. lottini L1* (N = 1 juvenile)	1							
		A. lottini L1*	*T. lutea L2*	1							
		—	—			2					
	*T. lutea L2*	A. lottini L1*	—	1				1			
		A. lottini L2*	—						1		
		—	—						1		
	*T. bidentata L2*	A. lottini L1*	—	1							
		A. lottini L2*	—					1			
	*T. serenei*	A. lottini L2*	—					1			
	—	A. lottini L2*	—			1					
	—	—	—			7				9	17
RI	T. guttata*	—	—		7		7				2
		A. lottini L1*	—		13		1		1		
			*T. bella*		1						
	T. speciosa*	A. lottini L1*	—		2		1		1		
		—	T. guttata*								1
			—				7		23		15
			*T. bidentata L1 + T. serenei*						1		
	*T. bella*	A. lottini L1*	—		2						
		—	—		1				2		
	*T. bidentata L1*	A. lottini L1*	—		1						
	*T. lutea L1*	A. lottini L1*	—		1				1		
		—	—				2		1		1
	—	A. lottini L1*	—		1						
	—	—	—		1		13		1		6

^*^exosymbiotic key species.
