# Erratum: Molecular characterization reveals the complexity of previously overlooked coral-exosymbiont interactions and the implications for coral-guild ecology

**DOI:** 10.1038/srep46818

**Published:** 2017-05-16

**Authors:** H. Rouzé, M. Leray, H. Magalon, L. Penin, P. Gélin, N. Knowlton, C. Fauvelot

Scientific Reports 7: Article number: 4492310.1038/srep44923; published online: 03 30 2017; updated: 05 16 2017

This Article contains typographical errors in Table 2. For Gene ‘COI’, species ‘*T. guttata*’ under ‘Present study’, the location ‘RI’ should read ‘RI, NC’.

