# Supplementary material for: Molecular characterization reveals the complexity of previously overlooked coral-exosymbiont interactions and the implications for coral-guild ecology
Source: Sci Rep. 2017 Mar 30;7:44923. doi: 10.1038/srep44923 (PMC5372162; doi:10.1038/srep44923)

SUPPLEMENTARY INFORMATIONS

Molecular characterization reveals the complexity of previously overlooked coral-exosymbiont interactions and the implications for coral-guild ecology

Rouzé H.[1,*], Leray M.[2], Magalon H.[3], Penin L.[3], Gélin P.[3], Knowlton N.[2], Fauvelot C.[1]

1: UMR ENTROPIE (IRD, Université de La Réunion, CNRS), Laboratoire d'excellence-CORAIL; centre IRD de Nouméa, BPA5, 98848 Nouméa Cedex, New Caledonia.

2: National Museum of Natural History, Smithsonian Institution, Washington, DC 20013, USA

3: UMR ENTROPIE (Université de La Réunion, CNRS, IRD), Laboratoire d'excellence-CORAIL; Université de La Réunion, 15 Boulevard René Cassin, CS 92003, 97744 Saint Denis, Reunion island.

* present address : Tropical Biosphere Research Center, University of the Ryukyus, Motobu, Okinawa, Japan,

Table S1

Pairwise genetic distance (mean) among the ten *Trapezia* lineages and the outgroup species (16S: *Quadrella maculosa* and COI: *Lybia* sp.) estimated using mtDNA 16S (below the diagonal) and COI (above the diagonal) partial genes. Species are indicated as follows: *Trapezia speciosa* (Tspe), *T. guttata* (Tgut), *T. septata* (Tsept), *T. cymodoce* (Tcymo), *T. bidentata* L1 (TbidL1) and L2 (TbidL2), *T. bella* (Tbel), *T. serenei* (Tser), *T. lutea* L1 (TlutL1) and L2 (TlutL2).

	**Outgroup**	**Tgut**	**Tser**	**Tbel**	**Tcymo**	**Tlut L1**	**Tlut L2**	**Tbid L1**	**Tbid L2**	**Tsept**	**Tspe**
**Outgroup**		0.208	0.178	0.190	0.155	0.181	0.176	0.205	0.196	0.197	0.190
**Tgut**	0.478		0.098	0.097	0.060	0.112	0.112	0.120	0.122	0.099	0.114
**Tser**	0.681	0.160		0.014	0.055	0.088	0.080	0.101	0.092	0.091	0.073
**Tbel**	0.674	0.165	0.004		0.058	0.095	0.086	0.106	0.099	0.100	0.074
**Tcymo**	0.595	0.191	0.161	0.151		0.055	0.045	0.071	0.074	0.055	0.062
**Tlut L1**	0.537	0.172	0.128	0.127	0.056		0.044	0.104	0.115	0.103	0.082
**Tlut L2**	0.490	0.150	0.126	0.116	0.055	0.026		0.110	0.111	0.084	0.081
**Tbid L1**	0.484	0.209	0.210	0.225	0.112	0.086	0.088		0.025	0.097	0.103
**Tbid L2**	0.434	0.179	0.161	0.174	0.104	0.061	0.072	0.021		0.095	0.099
**Tsept**	0.492	0.170	0.160	0.174	0.114	0.103	0.099	0.147	0.107		0.102
**Tspe**	0.781	0.185	0.116	0.108	0.132	0.172	0.172	0.212	0.152	0.136	

Table S2

Results of the Factorial Correspondence Analysis of exosymbiotic communities associated with *Pocillopora damicornis* types α and β (*sensu* Schmidt-Roach *et al.* 2014) at different ontogenic stages: adult versus juvenile on axes 1 and 2. Pearson product-moment correlation coefficient (*t*) measures the degree of linear dependence between variables and axes (values >1: positive correlation, values <1: negative correlation and values ~0: no correlation), and the p-value (*P*) indicates whether *t* is significantly different from zero.

**Stage**	**Variable species**	**Axis 1**		**Axis 2**	
		**t**	**p**	**t**	**p**
Adult df=118	Alot	0.3731	0.7097	-0.9586	0.3397
	Alot L1	10.5336	**<0.001**	-5.4095	**<0.001**
	Alot L2	-11.485	**<0.001**	-4.3822	**<0.001**
	Tbel	0.8622	0.3903	0.7631	0.4469
	Tbid L1	0.3488	0.7279	1.0615	0.2906
	Tbid L2	-0.2261	0.8216	-0.2074	0.8361
	Tcymo	-3.2008	0.001761	-1.0943	0.2761
	Tlut L1	0.7992	0.4258	-0.1043	0.9171
	Tgut	9.4209	**<0.001**	-2.1868	**<0.05**
	Tlut L2	-0.3971	0.692	-0.4136	0.6799
	Tsept	-5.8798	**<0.001**	-9.4758	**<0.001**
	Tser	-0.8206	0.4136	-0.6899	0.4916
	Tspe	-1.3862	0.1683	24.1969	**<0.001**
Juvenile df=112	Alot	NA	NA	NA	NA
	Alot L1	-0.105	0.9166	-0.7969	0.4272
	Alot L2	-0.0833	0.9338	-0.8502	0.397
	Tbel	NA	NA	NA	NA
	Tbid L1	NA	NA	NA	NA
	Tbid L2	NA	NA	NA	NA
	Tcymo	-0.13	0.8968	-1.4745	0.1431
	Tlut L1	-0.1546	0.8774	-1.666	0.0985
	Tgut	15.3869	**<0.001**	6.8283	**<0.001**
	Tlut L2	NA	NA	NA	NA
	Tsept	-0.3074	0.7591	-5.2301	**<0.001**
	Tser	NA	NA	NA	NA
	Tspe	-11.7312	**<0.001**	8.9274	**<0.001**

Figure S1

Phylogenetic tree of *Pocillopora damicornis* types α and β (*sensu* Schmidt-Roach *et al.* 2014) and others Pocilloporidae sequences derived from Bayesian approaches using sequences of the ORF region from this study (in bold) and incorporating published genetic sequences from GenBank (in regular font). Bayesian posterior probabilities (first values) and Maximum Likelihood bootstrap support values (in brackets) are presented. RI for Reunion Island, NC for New Caledonia. *Pocillopora*: Type α (*P. damicornis*), Type β; Type δ; Type γ (*P. verrucosa*); Type *e/m* (*P. eydouxi* and *P. meandrina*); Type ε (*P.* cf. *brevicornis*).

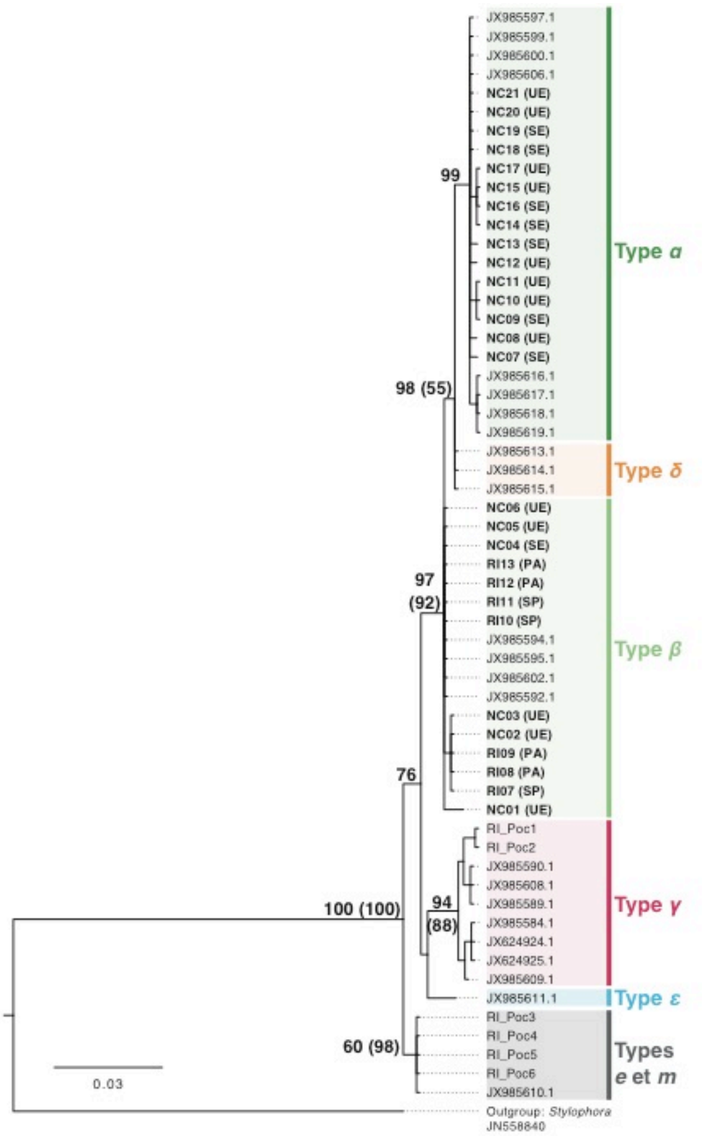

Figure S2

Pictures of *Trapezia* crab species identified from *Pocillopora damicornis* types α and β (*sensu* Schmidt-Roach *et al.* 2014) colonies from Reunion Island and New Caledonia.

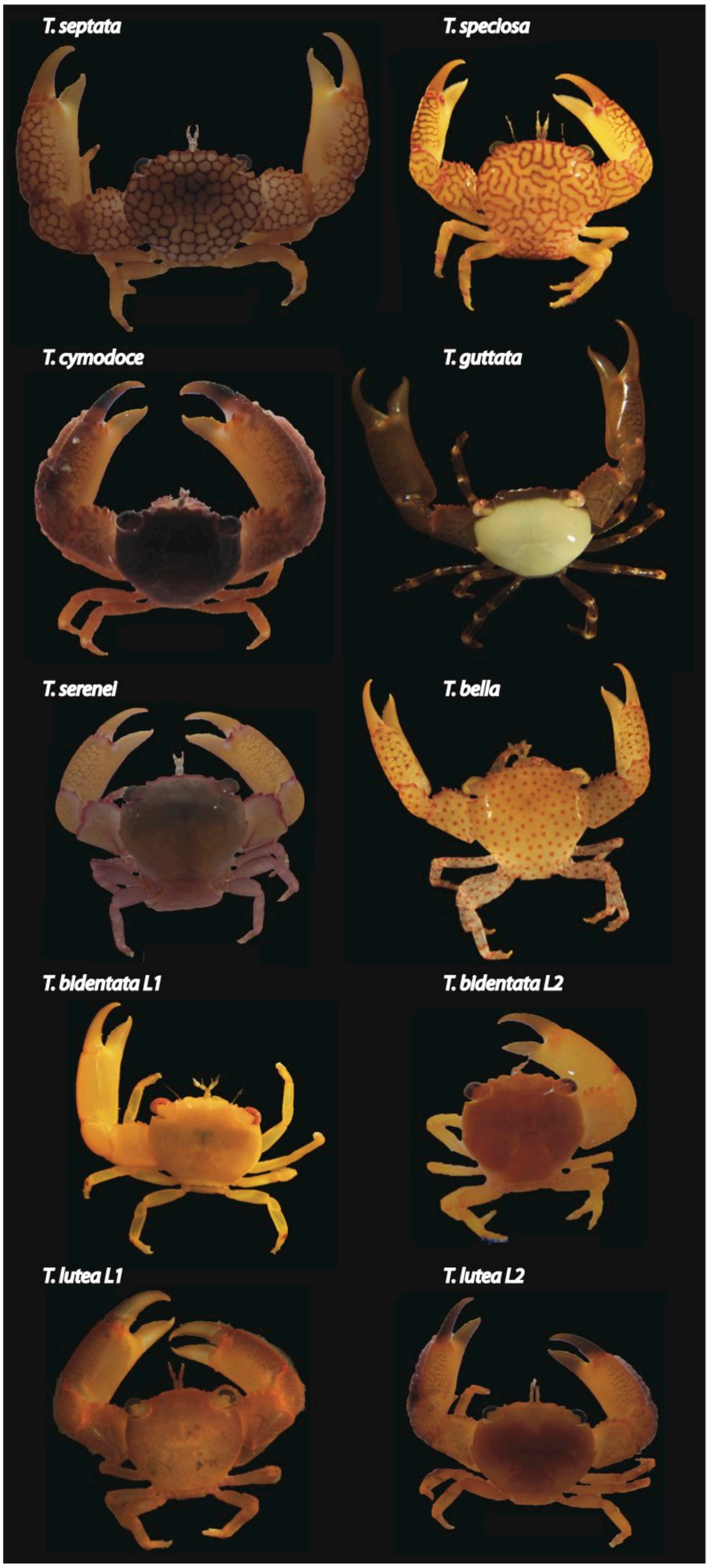

Figure S3

Frequency of mono-specific occurrence of the five exosymbiotic key species in adult *Pocillopora damicornis* type β from Reunion Island (RI) or from New Caledonia (NC), and type α from New Caledonia (NC). Black circles represent the observed data, the number of corals in which each species was observed alone. Colored rectangles represent the 95 % confidence intervals from 10,000 random simulations of mono-specific assemblages of shrimps (red) or crabs (blue) generated with the unconstrained null model M1, the 'frequency' constrained model M2 and the 'richness' constrained model M3 (see methods for a description of the models).

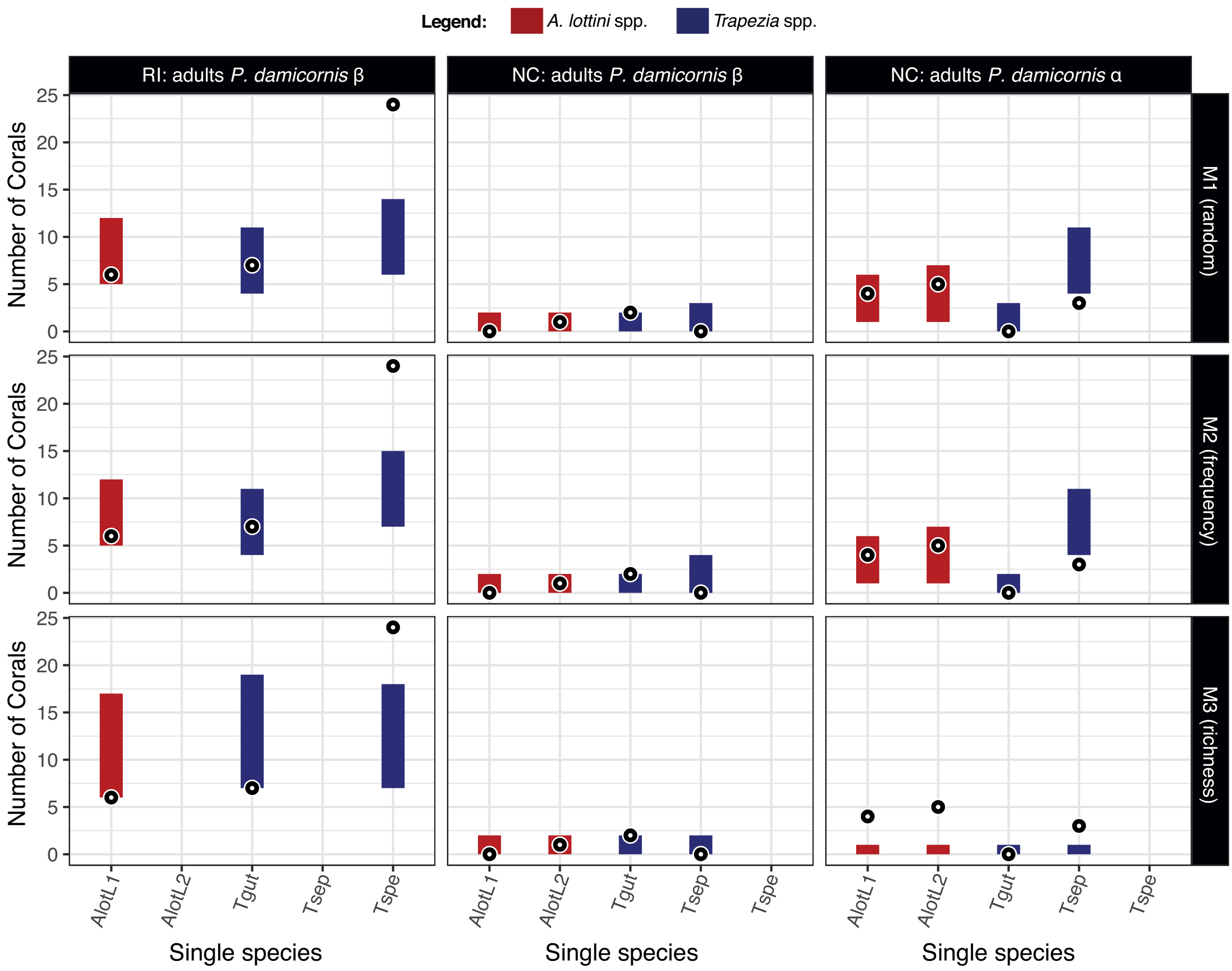

Supplement: Supplementary Information [file srep44923-s1.pdf]
